# Recycling of plaster of Paris

**DOI:** 10.4102/ajod.v9i0.503

**Published:** 2020-05-27

**Authors:** Servas Shiyo, Jozef Nagels, Harold G. Shangali

**Affiliations:** 1 Department of Prosthetics and Orthotics, Kilimanjaro Christian Medical Centre (KCMC), Moshi, United Republic of Tanzania; 2 Physical Rehabilitation Programme, International Committee of the Red Cross (ICRC), Geneva, Switzerland; 3 Faculty of Rehabilitation Medicine, Kilimanjaro Christian Medical University College (KCMU-College), Moshi, United Republic of Tanzania

**Keywords:** Recycling POP, calcination time and temperature, compressive test, setting time and reusability of POP, working properties of recycled POP

## Abstract

**Background:**

Plaster of Paris (POP) is being used in different ways in the field of medicine, dentistry and rehabilitation. One of its uses is in the manufacture of models of body segments in prosthetics and orthotics. It is used as a one-off procedure in which the used material is dismantled and discarded. The disposal of discarded materials does not allow easy decomposition which then pollutes the environment. It is not known whether this material could be reused if recycled.

**Objectives:**

The main objective of the study was to recycle POP models and determine its reuse in producing models with identical qualities, and thus reduce environmental pollution.

**Method:**

The procedure adopted was to break discarded models into small pieces, remove impurities and dirt; then the sample models were milled, washed, dried and pulverised. The POP models were heated to evaporate crystalline water in order to determine for how many times it could be recycled while retaining the desired strength, setting time and working characteristics.

**Results:**

The recycled POP reached higher setting temperatures and was stronger in terms of compressive strain and strength than the virgin POP. The highest temperature recorded for recycled POP was 40°C, which was higher than that for virgin powder (32.5°C). Testing compressive strength of all cylinders in all groups showed that the average compressive strength of the recycled powder mixed with water in a ratio of 1:1 was 2407 KN/m² and the ratio of 2:3 resulted in a compressive strength of 1028 KN/m², whereas the average compressive strength of virgin POP powder mixed with water in a ratio of 1:1 was 1807 KN/m² and the ratio of 2:3 resulted in a compressive strength of 798 KN/m². There were no differences in working properties between the recycled POP and the virgin POP.

**Conclusion:**

It was therefore concluded that under controlled conditions, such as grinding size, heating temperature, time and avoidance of contamination, used POP could be continuously recycled, resulting in stronger and workable casts.

## Introduction

Plaster of Paris (POP) came to be known as such because of the large gypsum deposits at Montmartre in Paris. It was also commonly called the gypsum plaster, produced by heating gypsum up to about 150°C in the presence of air. The heated gypsum, that is, calcinated and roasted, in which some water is lost as steam, contains only half the quantities of the water of hydration, which is called gypsum hemihydrate or beta hydrate.

The composition of gypsum-hydrated calcium sulphate (CaSO_4_.2H_2_O) is calcium 23.28%, sulphur 18.62%, hydrogen 2.34% and oxygen 55.76%. Calcium sulphate has been used wisely in several ways in the construction industry, agriculture, medicine, architecture and art. Sharp and Cork (2006) estimated that about 102 million tons of gypsum and anhydrite was produced in 2004. The production had grown to 250 million tons universally (Yu & Brouwers 2010).

### Gypsum hydration

Currently, three polymorphs of gypsum are well recognised; they are formed because of different preparation methods, crystal morphology, impurities and/or supplements/derivatives. Both α-hemihydrates and β–hemihydrates undergo hydration reactions in slightly differing mechanisms accompanied by exothermic changes. Addition of water to gypsum results in the formation of pastes with interlocking structures, which are responsible for gypsum setting strength (Singh & Middendorf 2007).

### Dehydration of gypsum

Vazquez-Almazan et al. (2012) indicated that when heated, 21% of water in calcium sulphate dehydrate (gypsum) undergoes dissociation from the mineral before evaporation, forming harder calcium sulphate hemihydrate. This process is now understood to be through endothermic decomposition reactions:

$${\text{CaSO}}_{4}.2{\text{H}}_{2}\text{O}+\text{heat}\to {\text{CaSO}}_{4}.0.5{\text{H}}_{2}\text{O}+1.5{\text{H}}_{2}\text{O}$$ [Eqn. 1]

$${\text{CaSO}}_{4}.0.5{\text{H}}_{2}\text{O}+\text{heat}\to {\text{CaSO}}_{4}+0.5{\text{H}}_{2}\text{O}$$ [Eqn. 2]

Yu and Brouwers (2010, 2012) reported that the amount of water needed for the hemihydrate is critical in the hydration reaction. The mechanisms of setting and hardening of gypsum plaster have been explained by the crystalline theory. Fine dehydrate is usually used to accelerate hydration to the desired setting time by changing the nucleation rates of generated dehydrate. Mechanically, the natural hardened gypsum has a high void level, consequently it is not a very compact solid. Gypsum strength evolves during setting because of rapid formation of interlocked matrix of β-dehydrate needles (crystals), followed by internal stress relief and removal of excessive water (Yu & Brouwers 2010).

However, there is no information regarding the recycling qualities of calcium sulphate used in waste generated from its use in prosthetics, orthotics, orthopaedics or dentistry. Wastes are in the form of improper/untimely setting of gypsum polymer and also from the gypsum polymer removed, for example after the repair of broken bone.

### Gypsum uses and applications

Abundant natural availability of gypsum and its easy response to water, heating and rehydration make it a popular choice in the construction industry. One of the advantages of gypsum is that it is not hazardous to humans and plants. Owing to its affinity to water, as a soil additive, gypsum improves soil physics and chemistry. It is an excellent source of calcium and sulphur for crop nourishment, especially in crops such as alfalfa, wheat, peanuts and cotton.

**FIGURE 1:**
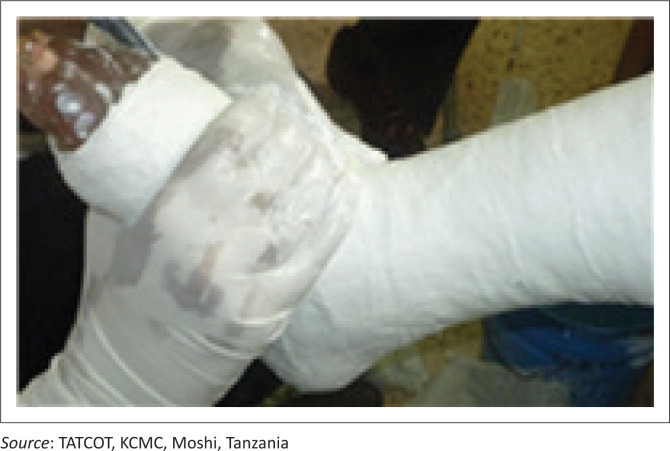
Manual casting of a leg with plaster of Paris (POP) bandage.

**FIGURE 2:**
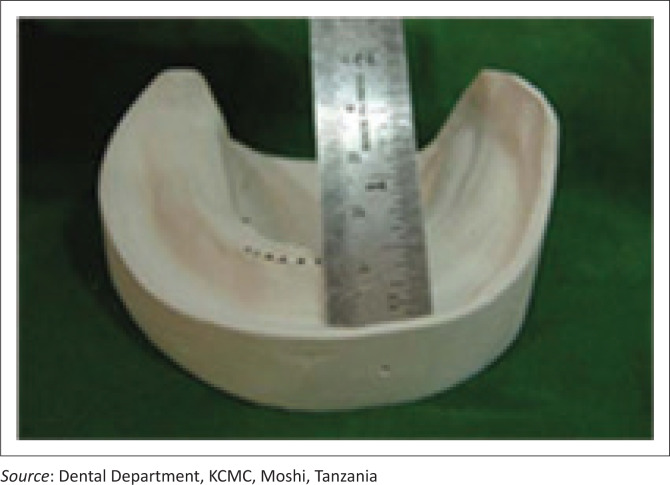
Positive cast made out of plaster of Paris (POP).

In medicine, it has been used widely as a support for fractures, that is, broken bones (Figure 1). When such a cast is applied to support and maintain the corrected position of a fractured segment of a bone, it is referred to as an orthopaedic cast. However, this is slowly being replaced by fibre glass. In dentistry, POP is used for mounting casts or models of oral tissues (Figure 2). This transfer of measurement and shape facilitates an optimum alignment of anatomical structure of the teeth and its configurations (Lokuliyana, Petera & Gunawardane 1988).

**FIGURE 3:**
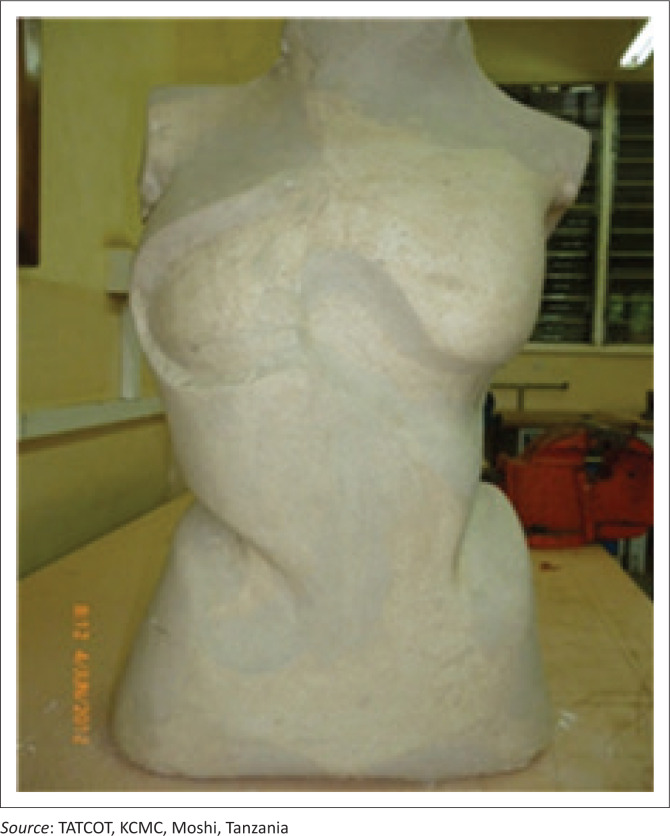
Plaster of Paris (POP) positive casts modified to fabricate spinal orthosis.

**FIGURE 4:**
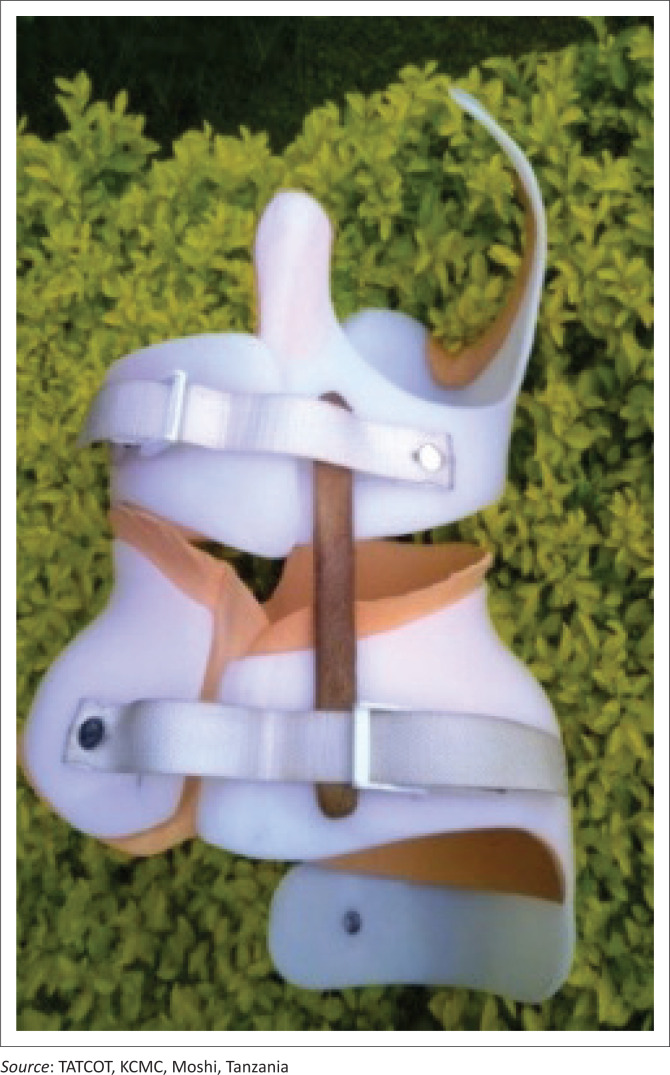
Spinal Orthosis molded on a plaster of Paris (POP) positive cast.

In prosthetics and orthotics, POP powder is used to produce positive casts/models for fabricating mobility-assistive devices. The process used is either through lamination or thermoplastic moulding. Figure 3 shows a rectified POP positive cast ready for moulding a thoraco-lumbar-sacral orthosis (Figure 4), which is used for treating idiopathic scoliosis.

Owing to its rapid setting properties, gypsum plaster casts may not be usable if the fractured bone is not properly positioned prior to application of plaster. In many situations, such casts become waste, thus necessitating the repeated preparation of POP powder.

Furthermore, huge quantities of plaster have to be used for plastering, and after the bone is set and rejuvenated, this hardened plaster has to be thrown away, thus adding further to waste with consequent burdens on the disposal of such POP plaster casts. The environmental burdens are in the form of limited landfill disposal facilities, and regulated permissions. At the same time, gypsum contributes to the emission of sulphur dioxide in the environment (Szpadt & Augustyn 1991).

Upon completion of the moulding process, the POP positive cast is often thrown out. However, the positive cast does not dissolve easily and therefore pollutes the environment. It becomes even worse when it is not broken into small pieces, as there are no guidelines or policies for disposing of such materials in the country.

### Cost of plaster of Paris

The production of POP in most developing countries is very minimal as there are either no companies or very few companies in operation. The production is not consistent as there is often a breakdown of machinery. The cost of POP and therefore the fabrication costs of mobility-assistive devices are high.

### Contribution to the field

This study contributes to achieving sustainable and environmental-friendly disposal in daily use and practice. It also initiates some challenges of developing waste gypsum recycling processes. This in turn decreases the pollution of the environment, reserves raw material (gypsum) and increases its availability for prosthetics and orthotics use.

The aim of the study was to assess the feasibility of recycling of POP casts to be reused to produce positive cast models in order to reduce the waste, minimise pollution and increase the availability of POP for use in prosthetics and orthotics field.

## Methodology

### Study design

This was an experimental laboratory-based study and a sequential convenient sampling method was used, whereby all POP positive casts which were produced by the students and academic staff at the Tanzanian Training Centre for Orthopaedic Technologist (TATCOT) during May 2016 were used.

### Sample and participants

The average amount of POP positive casts/models used by the students and academic staff during the 1 month was found to be about 245 kg (about five bags of POP weighing 50 kg each). The sample size was determined by using the following formula of which the level of precision was 0.05:

$$\begin{array}{l} n=\underline{N}\\ 1+N(e{)}^{2}\\ \text{As the amount of POP used per month at TATCOT is }245\text{ kg},\\ n=\underline{245}\\ 1+245(0.05{)}^{2}=151.9\text{ kg} \end{array}$$

Therefore, the sample size used for the project was 152 kg,

where *n* = sample size; *N* = population; 1 = desired confident level; *e* = desired level of precision.

The study was carried out at the Kibo Gypsum Manufacturing Company Limited, Moshi, Tanzania, as well as at TATCOT at the Kilimanjaro Christian Medical Centre and Arusha Technical College, Arusha, Tanzania.

### Data collection and analysis

The procedure adopted was to break models into small pieces, removing impurities and dirt, and then the samples were milled, washed, dried and pulverised. It included thermoplastic/thermosetting moulds using recycled POP positive casts. The setting time, temperature changes during the setting time and compressive strength were determined. The data were collected by using weighing scale, oven, thermometers, stop watch, camera and strength testing jigs. The data were analysed by using strength compression jig and computer spreadsheet program (MS-Excel).

### Recycling process

#### Converting the moulds or casts into powder

**FIGURE 5:**
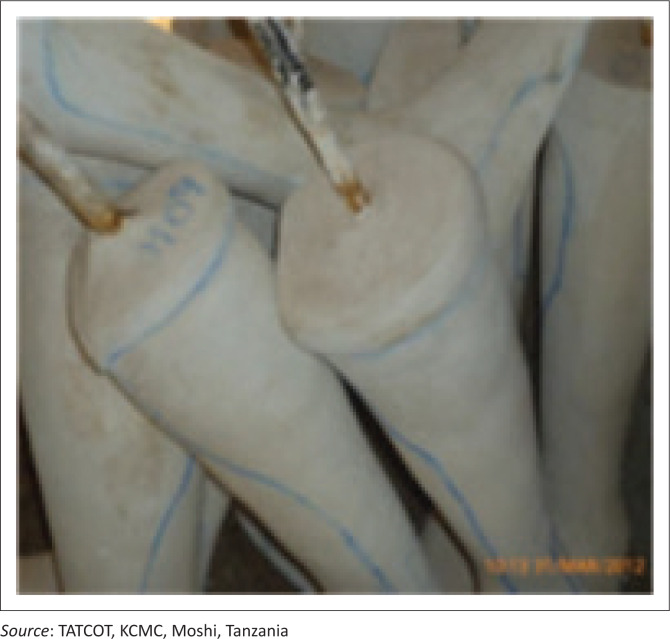
Plaster of Paris (POP) positive casts.

Dried POP positive cast models collected as seen in Figure 5 were produced by the students during their practical examinations. They were made out of similar negative models, which were used for all the students. These positive cast models were made from the same type of powder from the factory.

#### Calcination process

**FIGURE 6:**
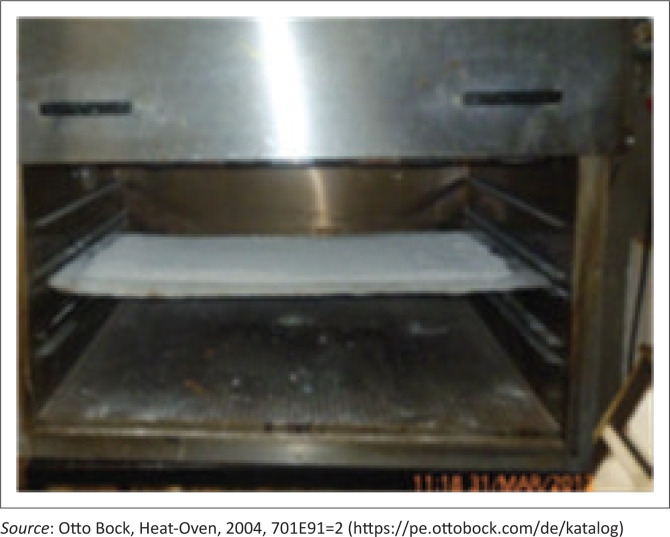
Heat oven for heating polypropylene plates used for molding different parts of assistive mobility devices.

Two kilograms of gypsum powder was kept in an oven (Figure 6) preset to 180°C for 2 h for calcination process and for determination of water of crystallisation.

#### Samples for compressive testing

**FIGURE 7:**
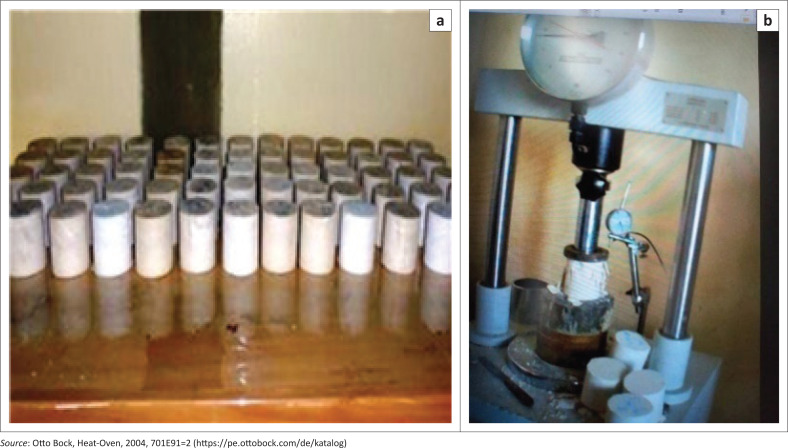
(a) Cylindrical plaster of Paris (POP) models for compressive testing; (b) A Germany Universal Testing machine (UTM), or materials test frame, is used to test the tensile strength and compressive strength of materials.

The samples reflected in Figure 7a were made out of polyvinyl chloride (PVC) pipes, which are cylindrical and have identical measurements and configuration. A mixed proportion of either 1:1 or 2:3 of water and gypsum powder was poured into the cylinders. They were later left to dry under the same temperature to ensure consistency for compressive testing for all the repeated recycled samples. The samples were made in a ratio of 1:1 which represented the equal mass of water and POP for one batch, and 2:3 in the other batch, which means that water was two-thirds of the POP used.

The compressive test was performed using the machine reflected in Figure 7b. The compression was maintained at a uniform velocity of 1 mm/min for all the samples. There were five cycles of compressive tests carried out on 12 samples in each batch, 6 for 1:1 and 6 for 2:3 ratio.

#### Packing

**FIGURE 8:**
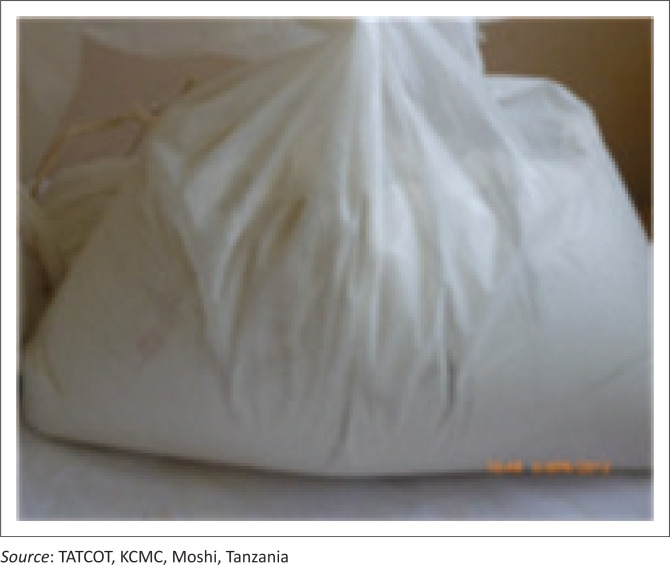
A Bag of plaster of Paris (POP) powder.

Owing to the hygroscopic property of POP powder when exposed to atmosphere, it reabsorbs water for crystallisation, thus reducing its reactivity power and strength. The calcinated POP powder has to be stored in an air-tight PVC bag for further use. Figure 8 shows the first recycled powder packed in a PVC bag and stored for further use. The recycled powder weighed 152 kg.

Ten kilograms of powder was set aside from each sample which was later used for comparison of its properties with the recycled and virgin POP powder samples. The remainder of POP powder was mixed with water to repeat the recycling process with recycled powder. The mixing of virgin POP with 1:1 and 2:3 ratio of water and POP powder respectively was repeated for six times. The second, third, fourth and fifth cycles with 1:1 and 2:3 ratio of water and POP powder respectively were also repeated for six times.

#### Preparation of plaster of Paris to recycle the powder

The same process was used in mixing the recycled powder with water to produce positive model casts for the second, third, fourth and fifth recycling rounds respectively. The casts were broken manually and later pressed through the hardened steel plates of a pressing machine. This resulted in the particles of the same size and volume. This was continued into a milling and calcination process. The milling wheel was controlled by cleaning after every three procedures to ensure that there was no dirt.

#### Weighing and standardising water and plaster of Paris powder

Water and POP powder were weighed on a scale to determine the mass of water and POP powder. Apart from using in-house drinking water and storing POP powder at room temperature, there was no other standardised procedure used.

#### Compressive strength of recycled plaster of Paris powder

**FIGURE 9:**
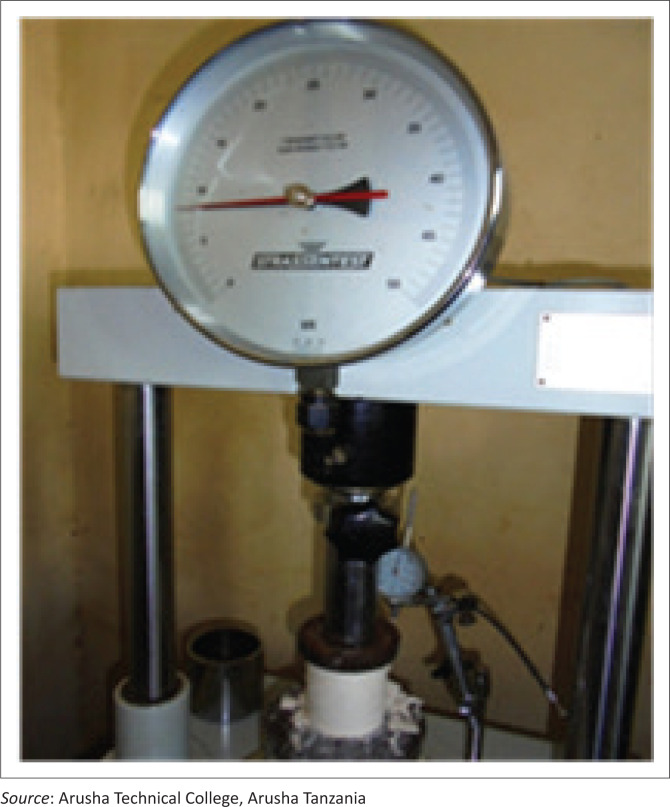
A Germany Universal Testing machine (UTM), or materials test frame, is used to test the tensile strength and compressive strength of materials.

A Germany-manufactured Universal Testing Machine ‘UTM’ was used for testing bricks (Figure 9). Fitted with perforated pelite on upper and lower surfaces, UTM was used to read and register data of strain and strength of different models tested. The two different mixing ratios of 1:1 and 2:3 of POP:water showed differences in the strain and strength of the models tested.

### Ethical considerations

Permission to carry out the study was obtained from Kilimanjaro Christian Medical University College (KCMU-College) of Tumaini University Makumira, Arusha, Tanzania (Certificate No. 735).

## Results

### Comparison of mass and temperature

The mass of POP positive casts was reduced by 17%, which reflected the amount of water of crystallisation that the gypsum powder contained as retained moisture.

The recycled POP mixed into two different ratios of 1:1 and 2:3 recorded a maximum temperature of 40°C and 36°C respectively, but the virgin POP mixed into two different ratios reached a maximum temperature of 33°C and 31°C respectively. The setting time of POP varies from 25 min to 60 min, depending on thickness.

**FIGURE 10:**
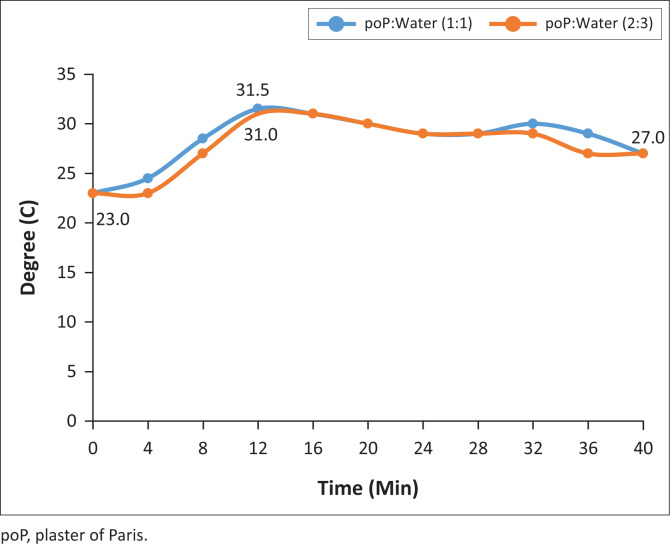
Virgin temperature changes.

**FIGURE 11:**
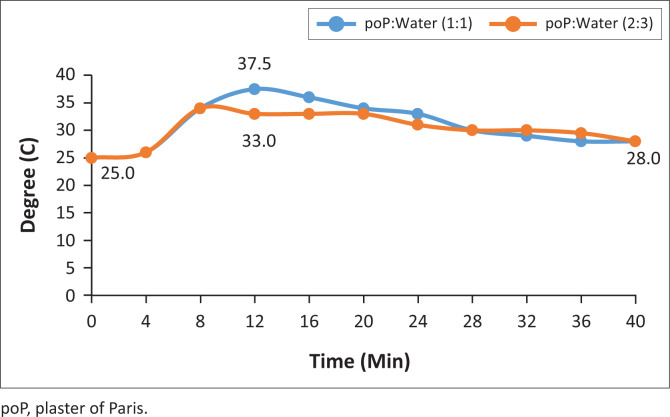
1st recycled temperature change.

**FIGURE 12:**
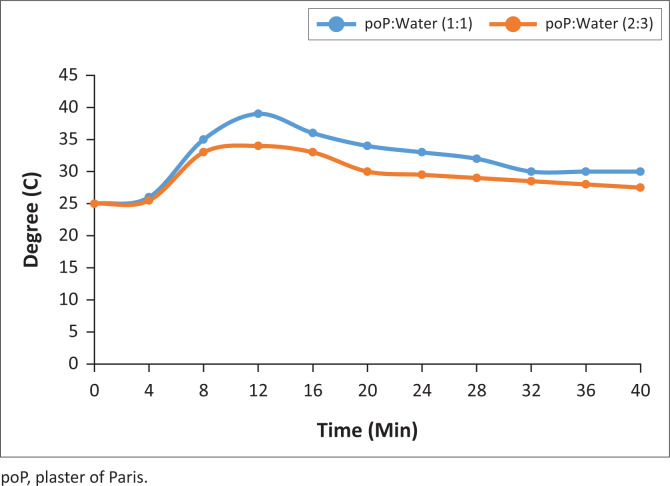
3rd recycled temperature changes.

**FIGURE 13:**
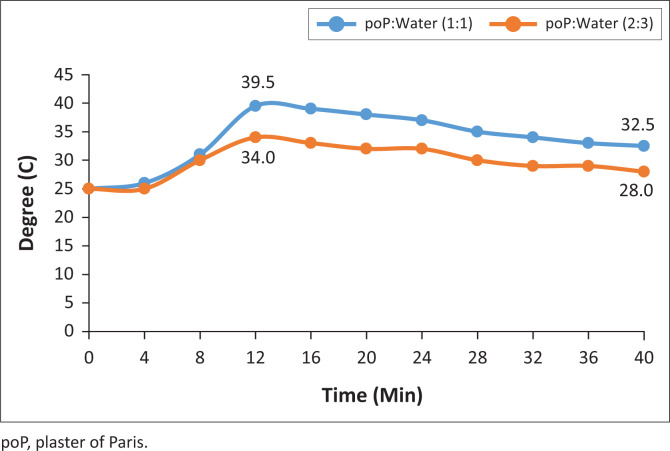
5th recycled temperature changes.

The graphs shown in Figures 10–13 indicate the setting time versus the temperature for four samples of the six systematically selected samples of POP mixed with water in two defined ratios (1:1 and 2:3).

The temperature patterns of three recycled POP samples are compared with the virgin POP extracted from initial casts. The temperature was recorded after time intervals of 2 min while observing the material’s behaviour with respect to its mixing ratio variants. The following are the vivid results obtained from the four graph patterns:

- The graph pattern of the virgin POP is identical to all the different levels of recycling, that is, 1st–5th level of recycling.
- In all graph patterns, the setting time starts as from the very first minute after mixing and is identical in both ratios.

The results indicate that the mixing ratios do not affect setting time but affect temperature released during the setting.

- After the initial 2 min mixing of POP, while the virgin POP reached an initial temperature of 23°C, those from the 1st, 3rd and 5th recycle reached the temperature of 25°C.
- There was a significant increase in temperature (8%) in the recycled POP sample (25°C) compared to the virgin POP sample (23°C), but the initial temperature was the same for all recycled POP samples.

In this study, the temperature pattern for all the samples was identical, and this is described by the initial, the highest and the lowest peaks of graphs. The setting time for all the samples was also identical, that is, 16th minute from the time of mixing.

The results shown in Table 1 reflect that there were rapid inclining trends of temperature towards the highest climax in all the trials. The mixing ratio was also significant in that the trial with ratio 1:1 showed higher temperatures in the last three trials. It was also evident that recycled POP starts with high setting temperature and ends with a higher setting temperature as compared to the virgin POP. The initial setting point for the virgin POP was 23°C and the highest was 27°C, while the initial setting point for recycled POP was 25°C and the highest point was 28°C.

**TABLE 1:** Increase in temperature with time and the peaks reached.

Recycling phases	Initial temperature (°C)		Highest peak temperature (°C)		Final temperature (°C)		Description of significance and variation
	1:1	2:3	1:1	2:3	1:1	2:3	
Virgin	23	23	31.5	31	27	27	The two mixing ratios had the same temperature for all the three levels.
1st recycle	25	25	39.5	33	28	28	The two mixing ratios initially had the same temperature but at the highest peak it showed a variation of 39.5°C vs. 33°C (16.5%) and at the final peak the temperature was the same.
3rd recycle	25	25	39	33	30.5	27.5	The two mixing ratios initially had the same temperature but at the highest peak it showed a variation of 39°C vs. 33°C (15.4%) and at the final peak a variation of 30.5°C vs. 27.5°C (9.8%).
5th recycle	25	25	39.5	34	32.5	28	The two mixing ratios initially had the same temperature but at the highest peak, it had a variation of 39.5°C vs. 34°C (13.94%) and at the final peak a variation of 32.5°C vs. 28°C (13.8%).

Plaster of Paris has two common forms, that is, the alpha hemihydrates and the beta hemihydrate. The alpha hemihydrate has a density of about 2.76 g/cm^3^, while the beta hemihydrate has a density of about 2.63 g/cm^3^. The estimated amount of water after drying was 25 g and 47 g for 1:1 and 2:3 mixing ratio respectively. This resulted in a density of 0.847 g/cm^3^ (1:1 ratio) and 0.637 g/cm^3^ (2:3 ratio), the 2:3 ratio revealing a lower density.

The POP mixed with water at 1:1 ratio retained higher mass weight relative to the POP mixed with water at 2:3 ratio.

Initially, all the sample models prepared from the same powder sample but mixed in two different ratios (1:1 and 2:3) had the same mass weight until setting process was complete, but they started losing excess water at different rates during dehydration of the model. The model from 1:1 ratio lost water slower than the model made from 2:3 ratio.

The two different mixing ratios of 1:1 and 2:3 of POP and water showed a significant effect on compressive strength, with recycled POP being 2.34 times stronger. For both virgin and recycled material, the higher the POP powder to water mixing ratio, the higher is the strength, and vice versa. This is because the powder crystals provide more sites for compact bond attachments that resulted in stronger structures by reducing porosities in POP mould.

While the average mass of the model made from recycled POP mixed at 1:1 ratio was 301.35 g, the virgin POP model had a mass of 287.92 g, showing a variation of 13.43 g (about 4.5%). The average mass of the model made from recycled POP mixed at 2:3 ratio was 226.0 g, while the virgin POP with a mass of 219.0 g shows a difference of 7.82 g (3.5%). In general, mass of the models made from POP mixed at 1:1 and 2:3 ratios were 299.11 g and 225.52 g respectively, which amounted to a difference of 73.5 g (approximately 24.25%).

**FIGURE 14:**
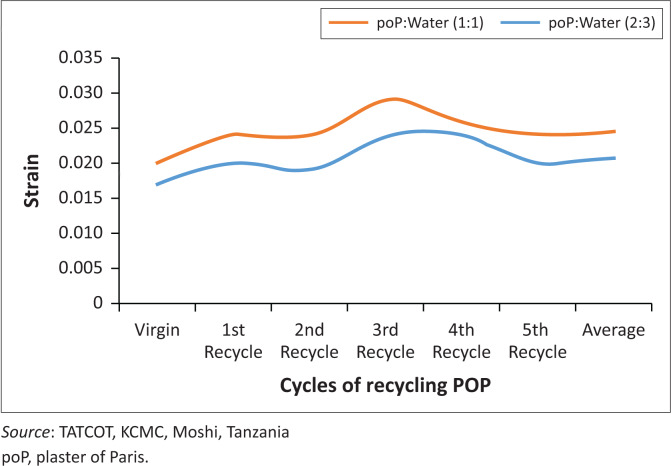
Plaster of Paris recycling trend.

Figure 14 indicates that, the model mixed at 1:1 ratio resulted in a volume change of 2.04%, while the model mixed at 2:3 ratio had a volume change of 2.42%. This indicated that there was 15.70% greater volume change with different ratio.

The recycled material showed the average highest compressive strength of 2407 kN/m² compared to the virgin material having an average compressive strength of 1807 kN/m². When the powder concentration is higher, the model was more compact and stronger with little deformation before attaining the ultimate point.

**FIGURE 15:**
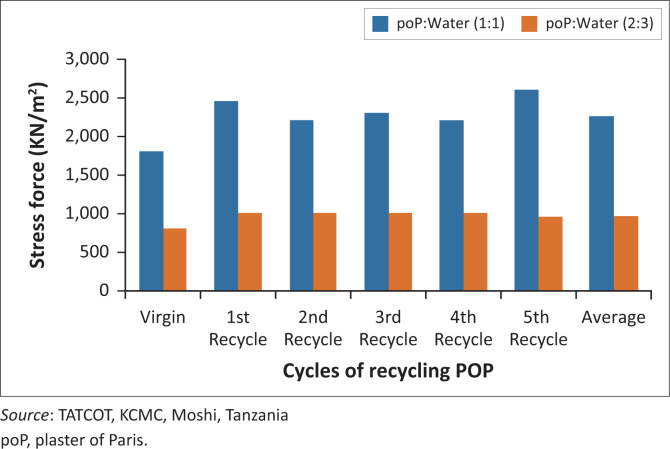
Compressive strength.

The compressive strength was affected by the mixing ratio of POP powder and water such that the average strength of the recycled powder mixed in 1:1 ratio was 2407 kN/m² whereas with 2:3 ratio it had an average strength of 1028 kN/m², and the virgin POP gave results of 1807 kN/m² and 798 kN/m² respectively. Therefore, the 1:1 mixing ratio yielded compressive strengths that were approximately 43% stronger than the 2:3 mixing ratio for both recycled and virgin powder (Figure 15).

### Workability of recycled plaster of Paris powder

For practical testing of the workability characteristics of recycled POP, two negative casts of bilateral ankle foot orthoses and one that of trans-tibia negative were taken by standard methods for filling with recycled POP slurry. Negative casts were removed after 1 h for rectifying and carrying out different procedures of thermoplastic and thermosetting of recycled POP with following observations.

Positive casts were of optimum standard which allowed for easy filing, nailing and cutting, and were able to accommodate the use of all the tools used for rectification, that is, modification of positive casts by using rasps (flat and half round). The impression of the force applied by virgin and recycled POP was similar. The nails applied during thermoplastic process were firmly held, indicating that the structure was hard enough. During moulding, recycled POP had an optimum strength to withstand the compressive force subjected to it during configuration and setting of plastic material either in the solid form or the liquid form, that is, when the pliers were used to mould a thermoplastic material, the plaster withstood tension without failure.

## Discussion

### Water of crystallisation

The degree of gypsum dehydration was strongly influenced by the material’s structure, particle size and impurities as well as by the conditions under which the process took place, such as temperature, heating rates, vapour pressure, humidity and particle size (Molony & Ridge 1968). In the same study, the average loss of water was 170 g and the temperature used was 180°C. The slight difference in the loss of water in which structures in the materials were not necessarily the same, was also witnessed by Molony and Ridge (1968).

### Compressive strength tests

Plaster of Paris can be produced by the heat treatment of discarded moulds under different conditions, and the quality of the product thus obtained depends on temperature and duration of burning. A temperature of 180°C with a heating time of 2 h was found to be the most suitable combination for conversion of used moulds into POP powder, which showed a compressive strength of 375 N (Lokuliyana, Petera & Gunawardane 1988).

### Workability of recycled plaster of Paris

There was no difference in the working properties of recycled POP and virgin POP. Mixing of recycled gypsum was much more economical because less powder was used to achieve and maintain the same strength as well as setting time of POP positive impression.

The physical and chemical properties of the product thus obtained were the same as those of commercially manufactured POP powder; therefore, the product obtained under these conditions could be used for manufacturing new moulds and also possibly as a cementation material for construction purposes (Lokuliyana, Perara & Gunawardane 1998).

### Environment conservation

Recycling used/waste POP reduces environment pollution as large quantities are recorded from waste products, while reduction of environmental waste has been strongly suggested, especially in mining processes. Friends of the Earth (2008) agree that recycling reduces the need for raw materials such as metals, forests and oil, and consequently reduces our impact on the environment.

### Cost of plaster of Paris

This study provided evidence-based facts to embark on recycling POP models and casts so as to avoid pollution and reduce the time spent and the costs of producing POP powder.

## Conclusions

Plaster of Paris can be produced by thermal treatment of recycled gypsum powder at a temperature of 180°C for 2 h. The results of this project show that POP could be recycled repeatedly with the same procedure without altering the required setting time and working characteristics of recycled POP powder for prosthetics and orthotics, and even improving the compressive strength of casts. Thus, recycling POP could preserve the environment and reduce pollution. It seems that recycling POP could reduce the cost of importing new POP. However, further study is needed to compare the costs of importing versus recycling POP.
